# Virtual student-led neuroscience conferencing: a UK multicentre prospective study investigating delegate outcomes and delivery mode

**DOI:** 10.1186/s12909-023-04779-z

**Published:** 2023-11-17

**Authors:** Emily R. Bligh, Yousif Aldabbagh, Jack Sheppard, Barbora Krivankova, Jay J. Park, Joe Cheung, Gergo Erdi-Krausz, Joshua Thomas, Hibatallah Altaher, Ellie Courtney, Tom Farrow

**Affiliations:** 1 Department of Neurosurgery, University Hospital Southampton, Southampton, UK; 2 Faculty of Medicine, Dentistry & Health, University of Sheffield Medical School, Sheffield, S10 2RX UK; 3 Insititute of Neurosciences, Glasgow, G51 4TF UK; 4 UCL Medical School, University College London, London, WC1E 6DE UK; 5 Department of Surgery, NHS Lothian, Edinburgh, UK; 6 School of Medicine, Dentistry & Nursing, University of Glasgow, Glasgow, G51 4TF UK; 7 Edinburgh Medical School, University of Edinburgh, Edinburgh, EH16 4SB UK; 8 Queen Mary University of London, Barts and The London School of Medicine London, London, UK; 9 Faculty of Medicine, University of Southampton, Southampton, SO17 1BJF UK

**Keywords:** Neurosciences, Neurology, Neurosurgery, Conference, Virtual, Education

## Abstract

**Background:**

Clinical neuroscience training programmes are becoming increasingly competitive to enter. UK university neuroscience societies act as a local environment for students to develop their career interests and provide portfolio building opportunities through hosting events such as annual conferences. Recently there has been a transition to more of these events being held online yet the impact of this, if any, remains unclear. This prospective study aimed to identify the impact of student-led neuroscience conferences on delegates and examine attitudes towards an online delivery approach.

**Methods:**

Multi-centre prospective survey study using pre-conference, post-conference, and 6-month post-conference online questionnaires distributed at 6 virtual student-led neuroscience conferences in 2021. The questionnaires had five-domains: demographics, career aspirations, academic skillsets, an educational manipulation check (EMC) and mode of delivery preference.

**Results:**

Nine hundred twenty-four surveys were completed across 559 conference attendances. 79.9% of delegates were medical students. Interest in a neuroscience career (*p* < 0.001), preparedness to undertake research (*p* < 0.001) and presentation (*p* < 0.001), as well as EMC scores (*p* < 0.001) increased immediately post conference. Most participants at 6 months post-attendance had completed an academic project (71.9%) or presentation (50.9%), although 88.8% were lost to follow up. Online format was preferred (65%) with reasons including elimination of travel and access to home facilities whilst lack of face-to-face interaction and engagement were recognised limitations.

**Conclusion:**

UK student-led online neuroscience conferences play a role in developing knowledge and may facilitate career interest, academic skillset and longer term portfolio building. A hybrid virtual and in-person experience would offer an ideal solution to future conferencing, providing options promoting engagement and interactivity whilst advocating sustainability, accessibility and widening participation.

**Supplementary Information:**

The online version contains supplementary material available at 10.1186/s12909-023-04779-z.

## Introduction

Clinical neuroscience training programmes are becoming increasingly competitive at national selection. In 2023, the speciality trainee (ST) competition ratio for neurosurgery ST1 and ST2 posts was 13:1 and 20:1 respectively, whilst a neurology ST4 post attracted 2.4 applicants per place [1]. High scoring applicants demonstrate clinical competence, a commitment to their desired specialty and offer a wealth of experience in academia, leadership and medical education [2, 3].

Moreover, there is reported fear towards neurosciences within the medical student community coined by the term ‘Neurophobia’ in 1994; alluding to some students struggling to engage or utilise their clinical neurology and neurosurgery knowledge [4]. Neuroscience societies at UK universities act as a supportive local environment for students to develop their neurology and neurosurgery career interests, interact with like-minded peers and provide students with the opportunity to attend annual events and conferences [5]. Whilst conference attendance does not contribute directly to application points per se, delegate activity during and leading up to the event such as; attainment of an oral or poster.

presentation for their academic work, winning an associated prize, or demonstrating leadership as an organising committee member, can be attributable to shortlisting points at national selection [6]. Furthermore, student-led events offer delegates the opportunity to network and grasp a deeper understanding of the neuroscience specialities, which may help delegates confirm or defer against a career in the clinical neuroscienceses [7]. Previous single centre research has indicated that in-person and virtual undergraduate neurosurgery conferences and careers days; strengthen career interest and exposure, build career knowledge and develop academic skills [7–10]. Yet, these results are largely based on delegate perceptions, specific only to a neurosurgical career, and there is no reproducible, standardised and objective outcome data.

The COVID-19 pandemic initiated a transition in conference delivery from in-person to online [11]. Although in-person conferences have been re-introduced, some student societies have opted to maintain an online format and there is scope for virtual events to widen participation to students from a low-socioeconomic background [12]. However, published literature examining the advantages, disadvantages and preference towards this delivery approach in a student population is scarce.

As such, this prospective multi-centre study holds two main objectives:

1. Identify the impact of online student-led neuroscience conferences, specifically through: knowledge built, interest in a neuroscience career and attainment of further research and presentation experience.
2. Explore the favourability, benefits and drawbacks of a virtual delivery approach.

## Methods

### Study Design

We prospectively surveyed delegates attending 6 virtual student-led neuroscience conferences in the UK from February 28th 2021 to April 24th 2021. The neuroscience societies involved in this study were Sheffield Neuroscience Society, UCL (University College London) Surgical & Medical Societies, Barts and the London Neuroscience Society, Glasgow Neuro Society, Edinburgh University Neurological Society and Southampton University NeuroSoc. All societies receive annual funding from their associated Medical School, university, or from individual fundraising activities and sponsorships.

Delegates filled out a pre-conference, immediately post-conference and 6 months post-conference questionnaire (Additional file 1, 2, 3) via Google Forms. The five-domain survey consisted of questions that determined demographics, career aspirations, academic skillsets, an educational manipulation check (EMC) and mode of delivery preference. The questionnaire consisted of Likert 10-point closed ended questions, multiple choice questions and multiple-choice check box ‘tick all that apply’ questions. All of these questions were standardised per unit, apart from the EMC which was 4 questions based on career, academic and neuroscience knowledge specific to individual conference content. The purpose of the EMC is to objectively assert the knowledge built. This is done by testing the delegates knowledge of lecture content before and after their teaching and assessing for any improvement. The questionnaire was developed by study authors (EB, JT, EC, JP, YA, JS) and modified following a pilot at the University of Sheffield Neuroscience Annual Conference in 2020. Initially a focus group of neuroscience committee members were utilised to elicit common multiple-choice answers for the questionnaire, as well as using similar studies for comparison points, these points in addition to an ‘other’ option were piloted in 2020. Following this, further themes were identified, and the response list was adjusted accordingly before final review and amendment by the national steering committee. A unique code linked participants to their subsequent responses.

All registered attendees were emailed with a link to the survey which, upon completion, would allow them access to the event. Post-conference surveys were sent out to delegates directly after the conference and closed 2-weeks post-conference with completion providing a link to their attendance certificate. The final survey was distributed via email to all previous survey participants at 5 months post-conference and closed at 7 months post-conference.

### Conference setting

All conferences were held exclusively online, had a neurology and neurosurgery focus and provided opportunities for delegates to present their own research (3/6 offered oral presentation only). The total delegate attendance was approximately 616 across the 6 conferences, with a mean average of 102 attendees per event (range 40 to 150). Five centres held their event over one day and the remaining centre held their conference over two days. Two thirds of the centres offered free admission whilst the other two centres charged between £3 and £5 per ticket with a discount available for widening access scheme students. All but one centre held a workshop. The keynote speakers and lecture content differed between conferences; however, all centres offered learning opportunities within clinical neurosciences, academia and career path expectations.

### Participants

All delegates attending the conferences included in this study were eligible to participate. There were no exclusion criteria based on stage in training or place of study/work. A small cohort of sixth form pupils enrolled in a widening access scheme were excluded at the Sheffield Neuroscience Society conference due to involvement in a separate study.

### Ethics

 Ethical approval was attained through the University of Sheffield Ethics Committee (Additional file 4). A participant information sheet including a General Data Protection Regulation (GDPR) statement was included in the questionnaire (Additional file 5) and all responses were made anonymous.

### Data analysis

Data were stored in Google Forms, Edexcel, and SPSS. Data were analysed with SPSS Version 28 and a significance level was set at 95%. Descriptive analyses and Wilcoxon Matched Pairs Tests were performed. Missing survey responses were excluded from all analysis. All those lost to follow up were not included in the prospective analysis. There was no comparison performed between centres.

## Results

Nine hundred twenty-four surveys from 435 delegates across 559 conference attendances were received. There were 511 pre-conference, 357 immediately post-conference and 57 six-month post conference responses.

### Delegate demographics

**Fig. 1:**
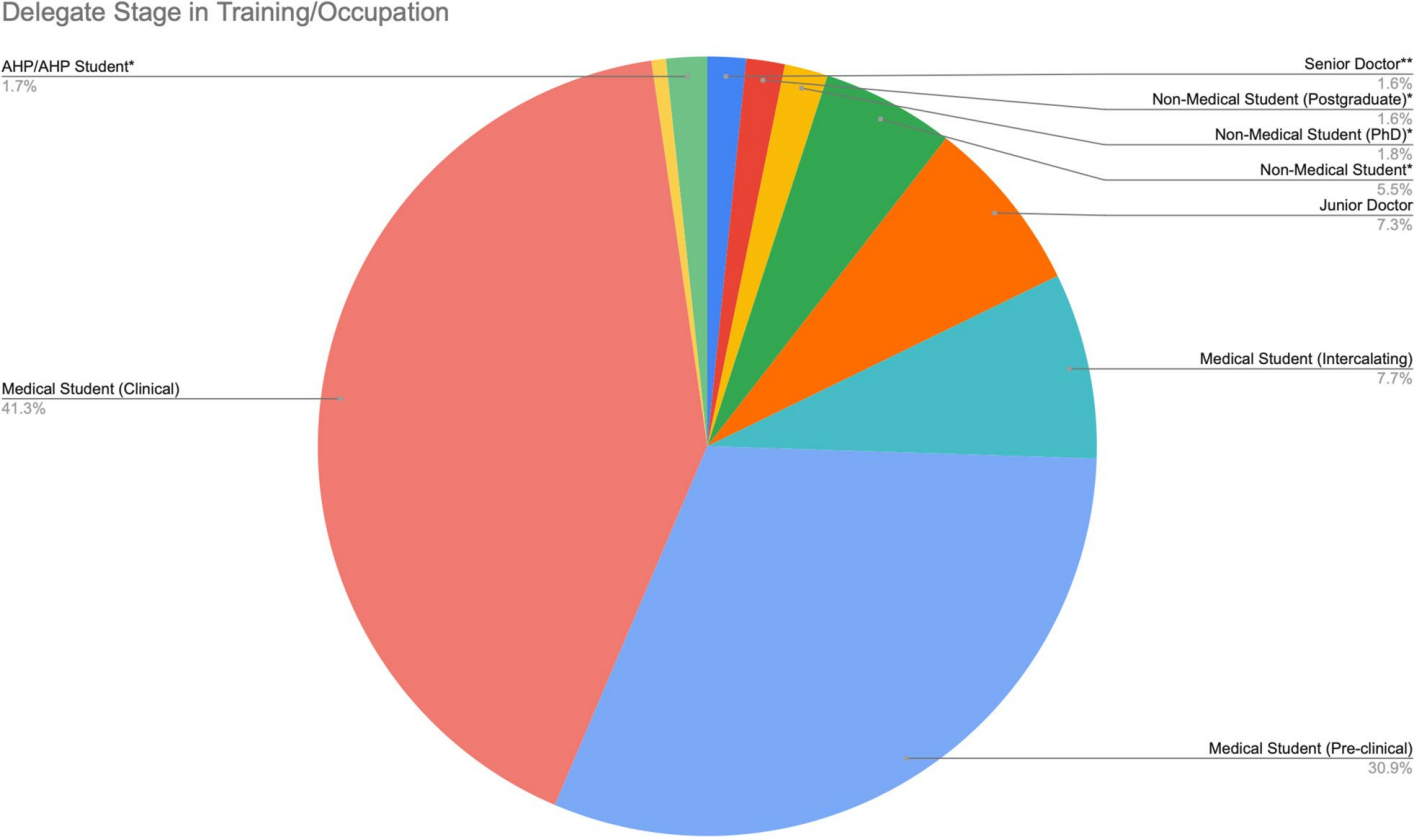
Participant stage in training/occupation. [* These options were not provided and were entered by participants in the ‘Other’ option. **A senior doctor was defined as a consultant or senior registrar]. The unlabelled slice refers to ‘Other’ of those that could not be grouped together. Number of respondents = 508

 Figure 1 demonstrates participant stage in training with the large majority indicating they were medical students (79.9%) whilst other attendees included non-medical students (8%), doctors (8%) and allied healthcare professionals (0.9%). 66.5% of delegates had never attended a student-led neuroscience conference before. Sixteen attendees had a confirmed oral presentation, whilst 15 had a poster presentation.

Attraction to the conference, previous portfolio building activity and neuroscience career interest are listed in Table 1. The most common reason for conference attendance was ‘Keynote speakers’ from 66.9% of respondents, followed by workshops (62.8%), opportunity to boost CV (44.6%) and opportunity to network (33.9%). Only 30/511 respondents were attracted to the conference with a goal to win a prize.

**Table 1:** A table demonstrating delegate demographics

Delegate Demographics (Tick all that apply)	Number of Responses	Proportion of Respondents
**Attraction to the conference**	1389	/511
Opportunity to present	55	10.8
Opportunity to network	173	33.9
Opportunity to win prizes	30	5.9
Opportunity to boost CV	228	44.6
Keynote Speakers	342	66.9
Other Speakers	146	28.6
Workshops	321	62.8
Institutions affiliated with our speakers	94	18.4
**Previous portfolio building activity**	534	/451
Neuroscience elective	31	6.9
Neuroscience SSC	60	13.3
Conference presentation	86	19.1
Neuroscience society committee member	57	12.6
None of the above	300	66.5
**Neuroscience career of interest**	639	/511
Neurosurgery	300	58.7
Neurology	271	53.0
Psychiatry	22	4.3
Neuroscience research*	8	1.6
Other	19	3.7
None	19	3.7

^*^These options were not provided and were entered by participants in the ‘Other’ option

Median interest in a neuroscience career was 8/10 pre-conference (mode:10, range: 9, IQR: 7–10). The majority of participants were interested in neurosurgery (58.7%) followed by neurology (53.0%). Other neuroscience career interests included psychiatry (4.3%) and research (1.6%). When asked ‘Do you experience neurophobia?’ 29.4% felt they did (6.5%) or might (22.9%).

When assessing previous experiences, the majority of those surveyed (66.5%) reported that they had no previous experience in building a neuroscience Curriculum Vitae (CV). 35.2% had previously undertaken their own neuroscience-related research project. Approximately one in five (19.1%) had given a conference presentation before, 13.3% had acted as a local neuroscience committee member and 12.6% had undertaken a neuroscience student selected component (SSC). Median preparedness to undertake a research project and presentation pre-conference were 6/10 (range: 10, IQR: 4–8) and 5/10 (range: 10, IQR: 4–8) respectively.

### Post-conference outcomes

**Fig. 2:**
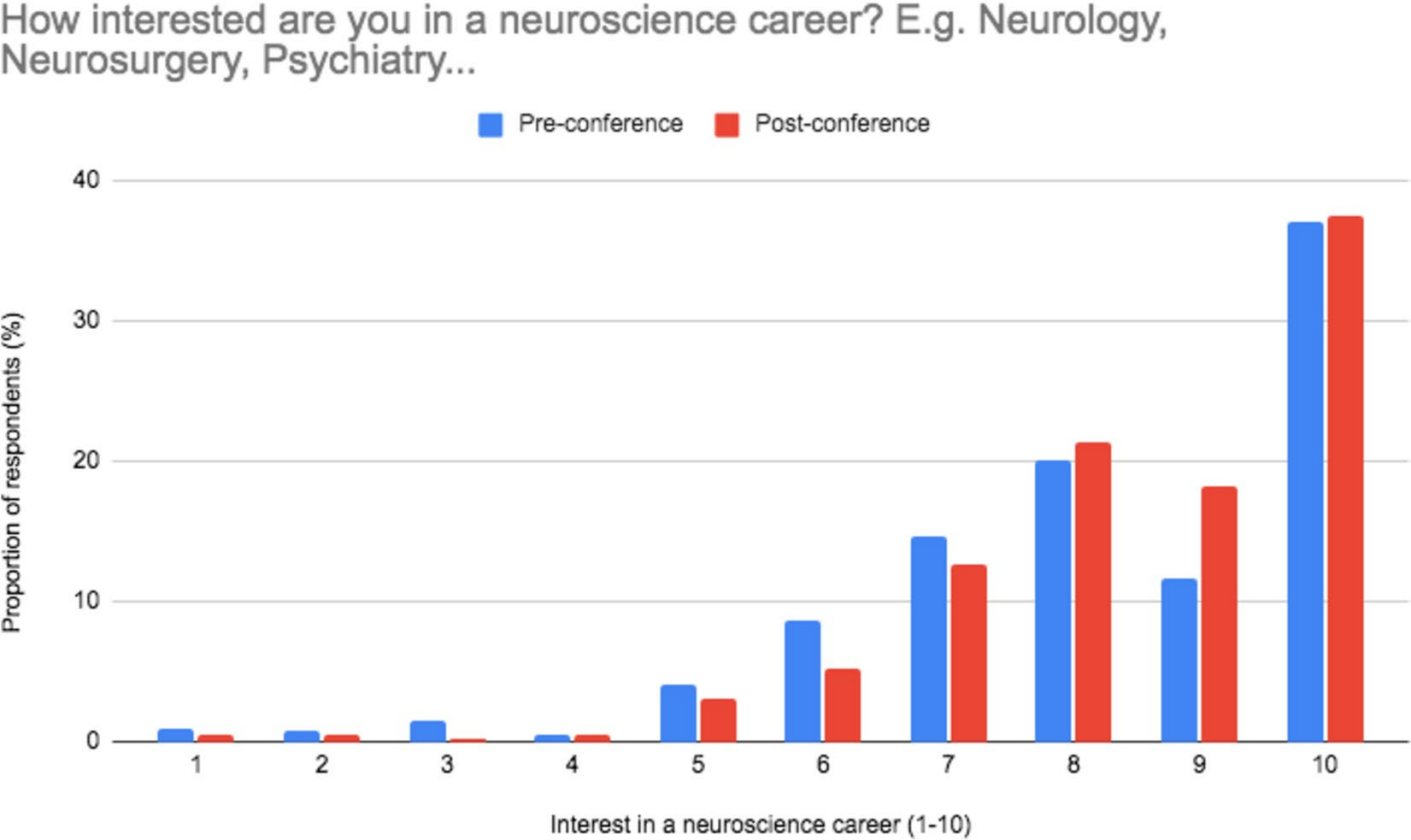
Participant responses to ‘How interested are you in a neuroscience career? E.g. Neurology, Neurosurgery, Psychiatry...’ before and immediately after the conference. 1=Least interested. 10=Most interested

 Matched pairs testing denoted that median neuroscience career interest increased significantly from 8/10 to 9/10 (*p* < 0.001), with a bar chart to demonstrate this shown in Fig. 2. Preparedness to undertake a research project increased from 6 to 7 (*p* < 0.001) and preparedness to carry out a presentation increased from 5 to 7 (*p* < 0.001).

EMC scores rose from a median of 2/4 (range 0–4) to 3/4 post-conference. This was found to be statistically significant when matched pair testing was performed (*p* < 0.001).

Delegates perceptions of their ‘most valuable part of the conference’ and ‘type of neuroscience career building activity they are inspired to participate in’ are shown in Table 1. Immediately post-conference, delegates felt that the most valuable part of the conference was ‘key notes speeches’ (77.3%), followed by ‘workshops’ (68.9%) and ‘opportunity to boost CV’ (44.8%). These 3 most frequently recorded answers also corresponded with the top 3 reasons for attraction to the event. Responders found the ‘opportunity to win prizes’ (28 responses) the least valuable part of the conference which correlates to the small proportion of delegates with a confirmed presentation at their respective conference.

**Table 2:** A table demonstrating delegate post-conference responses

Post Conference Responses (Tick all that apply)	Number of Responses	Proportion of Respondents
**Most valuable feature**	1020	/357
Opportunity to present	51	14.3
Opportunity to network	97	27.2
Opportunity to win prizes	28	7.8
Opportunity to boost CV	160	44.8
Keynote Speakers	276	77.3
Other Speakers	109	30.5
Workshops	246	68.9
Institutions affiliated with our speakers	53	14.8
**Inspired to do the following portfolio building activity**	1032	/306
Neuroscience research project	207	67.7
Neuroscience conference	194	63.4
Conference presentation	186	34.6
Neuroscience society committee member	129	42.2
Neuroscience elective/SSC	138	45.1
Further CV building activity	176	57.5
None	2	0.7

The neuroscience activity participants were most commonly inspired to pursue was a neuroscience research project (67.7%) followed by another neuroscience conference (63.4%). Less than 1% of attendees felt that the conference had not inspired them to complete any further career building related activity.

### Prospective outcomes

Median interest in a neuroscience career (9/10) and preparedness to undertake a research project (7/10) scores were entirely retained from the immediately post-conference scores. Median preparedness to undertake a presentation further increased from 7/10 to 8/10, however this rise was not found to be statistically significant after matched pair testing (*p* = 0.738).

**Fig. 3:**
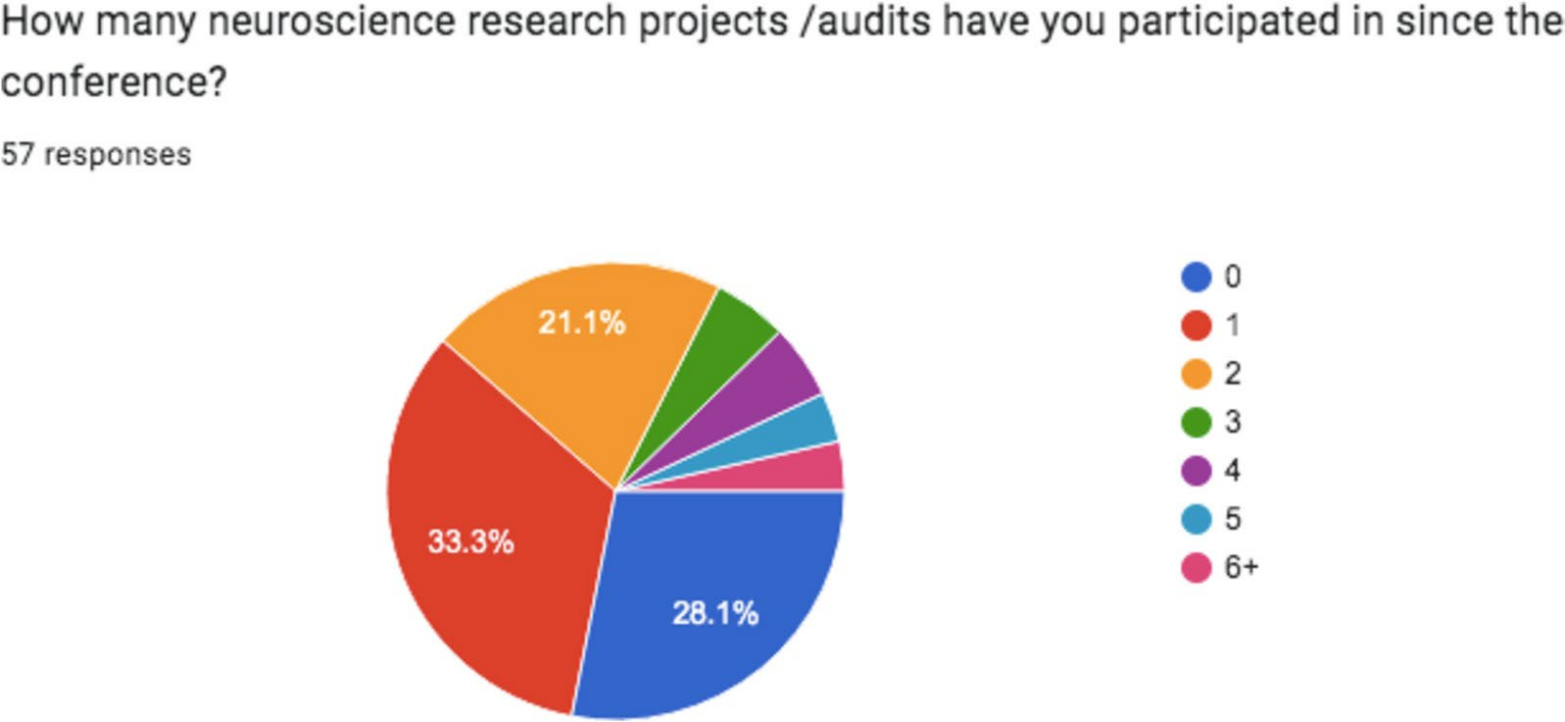
Participant responses to ‘How many neuroscience research projects /audits have you participated in since the conference?’. Number of respondents = 57

 Figure 3 demonstrates the number of neuroscience research projects/audits participants had undertaken in the post-conference period with 41/57 (71.9%) taking part in one or more of these activities. Over half (50.9%) of those surveyed had undertaken a conference presentation since the conference and 17/57 (29.9%) had completed two or more.

Other ways that delegates chose and/or intend to develop their neuroscience career interest are exhibited in Table 1. Since their initial conference attendance 6 months previously, 59.6% (34/57) had attended another neuroscience-themed conference, 49.1% (28/57) had gained involvement in a neuroscience society and 40.4% (23/57) had acquired teaching experience.

**Table 3:** A table demonstrating delegate 6-month post-conferencen responses

Prospective Conference Responses (Tick all that apply)	Number of Responses	Proportion of Respondents
**Activities completed since conference attendance**	122	/57
SSC	10	17.5
Elective	15	26.3
Neuroscience conference	34	59.6
Involvement in a neuroscience society	28	49.1
Teaching	23	40.4
Other^a^	10	17.5
None	2	3.6
**Activities confirmed at a later date**	127	/50
Presentation	17	34
Research project	35	70
Elective	16	32
Neuroscience conference	23	46
Neuroscience society	15	30
Teaching	18	36
Other^a^	2	4
None	1	2

^a^These options encompassed a variety of responses that were entered by participants via short answer text

The most common activity participants had confirmed to be undertaken at a later date was a research project (70%) followed by a neuroscience conference (46%), teaching (36%) and a presentation (34%). Only 1/57 indicated they had no confirmed activities from the multiple-choice option list.

Qualitative analysis of answers to ‘What role has the conference played, if any, in facilitating your neuroscience career related activity?’ highlighted a number of benefits indicated by participants. Five main themes arose including ‘knowledge developed’, ‘increased interest’, ‘career guidance’, ‘networking’ and ‘encouragement for further opportunities’.

### Mode of delivery

65% of delegates answered ‘Yes’ when asked ‘Do you prefer neuroscience conferences to be held online?’ immediately post-conference with the remaining 35% responding ‘No’.

**Fig. 4:**
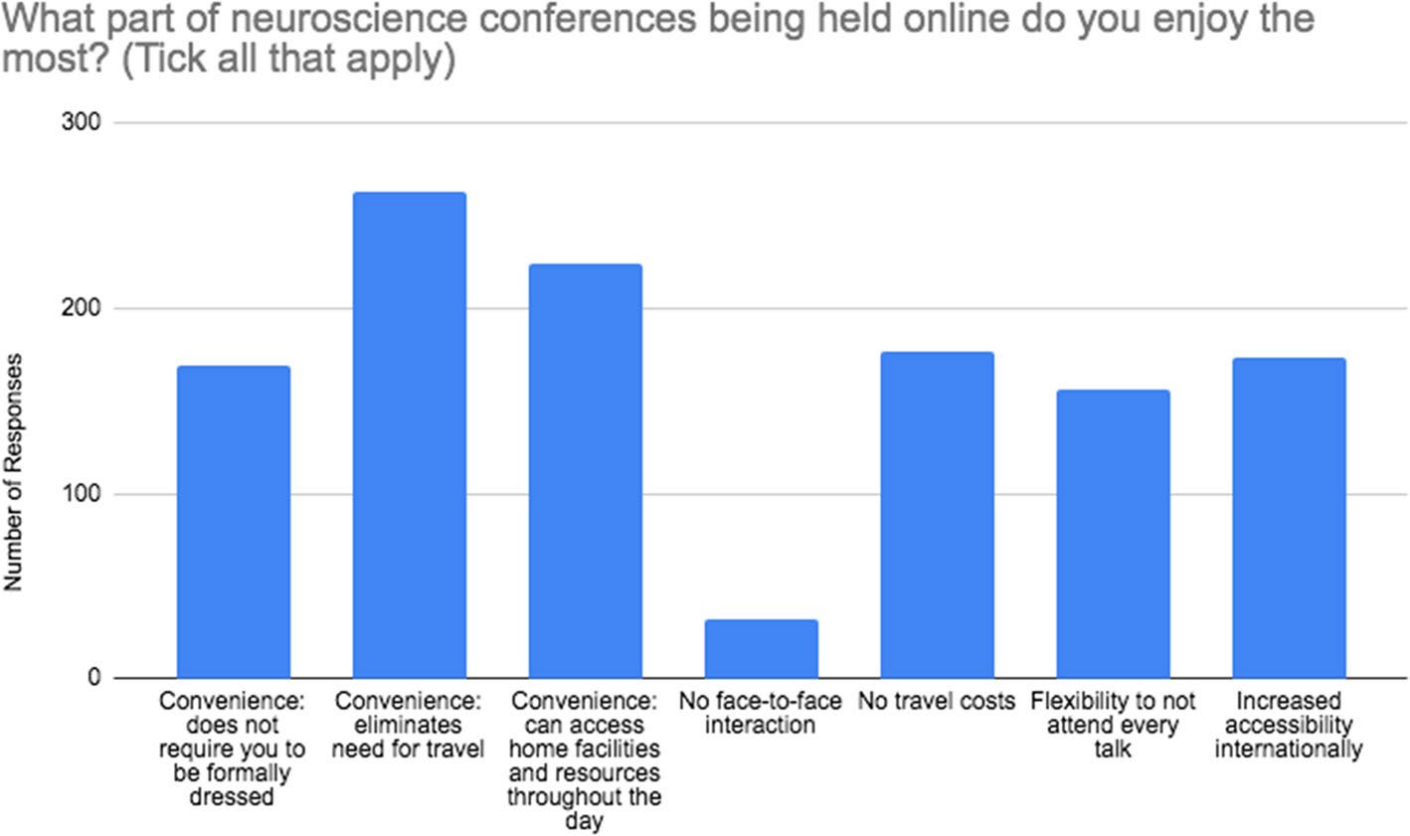
Participant responses to ‘What part of neuroscience conferences being held online do you enjoy the most? (Tick all that apply)’. Number of respondents = 306

**Fig. 5:**
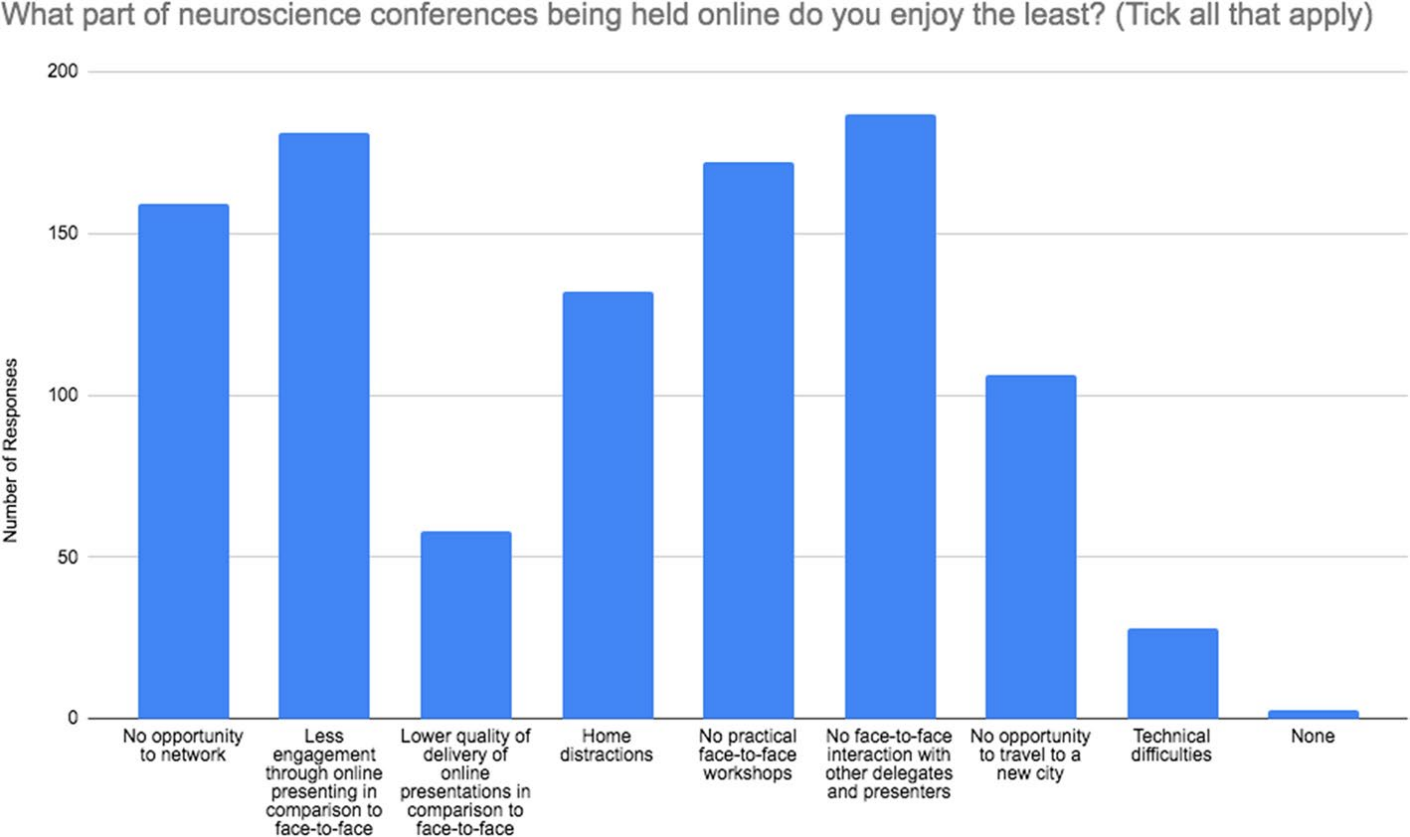
Participant responses to ‘What part of neuroscience conferences being held online do you enjoy the least? (Tick all that apply)’. Number of respondents = 306

 Figure 4 demonstrates the reasoning behind what delegates enjoyed the most about an online format whilst Fig. 5 shows responses to what they enjoyed the least. The most frequently chosen benefit of online conference delivery was elimination of the need for travel (85.9%) followed by access to home facilities (73.2%) and no travel costs (57.8%). Only 10.5% of responders felt that no face-to-face interaction made the conference more enjoyable.

Delegates least enjoyed having no face-to-face interaction with other delegates and presenters (61.1%), less engagement through online presenting in comparison to face-to-face (59.2%) and no practical face-to-face workshops (56.2%). Interestingly, technical difficulties and lower quality of online presentation delivery, in comparison to face-to-face, were selected by 9.1% and 19.0% of participants respectively.

At 6 months, participants were asked ‘Would you like future conferences to be virtual or in person?’ and were offered the options ‘Virtual’, ‘In Person or ‘Both’. 75% opted for both, 14.3% chose virtual only and the remaining 10.7% chose in person.

## Discussion

This analysis is the first multi-centre, large cohort, prospective study to evaluate student-led conferences and their impact on student to professional development. Our data reveals novel findings demonstrating an increase and retainment of career interest, preparedness to undertake research and presentations, as well as participation in extracurricular activities following their initial attendance at a neuroscience conference. Furthermore, our results confirm a significant increase in EMCs post-conference and thus being an effective information delivery platform to build delegate knowledge with positive feedback and an urge to continue a virtual approach.

Most respondents in our study were medical students who had not attended neuroscience conferences before, nor had they participated in research projects or further career building activity such as electives, student selected components, or becoming a member of a local neuroscience society. Single centre research by Hanrahan et al., also found that the majority of attendees are medical students who haven’t previously attended neuroscience conferences or taken part in related extracurricular activities [7]. This suggests that student-led virtual neuroscience conferences could act as an initial opportunity and gateway, allowing medical students to become engrossed in the field and facilitate decision making as to whether a clinical neuroscience career is suitable for them. This theory is reinforced by many of our small prospective cohort attaining neuroscience academic portfolio building opportunities post-conference. Given that respondents reportedly chose career building activity as a common reason for attending the online conference, our prospective data suggests delegates are able to meet this desired objective through attendance. Although, our loss to follow up of 88.8% should be considered when reviewing these results, an alternative explanation is delegates who attained further opportunities were also more likely to fill out our 6-month survey.

Our results indicated students were attracted to conferences by keynote speakers, workshops on offer and the opportunity to develop their CV. Follow up of students post-conference corroborates this, with attendees finding keynote speakers and workshops most valuable. In contrast, Al Omran et al. found that the most common reason for students to attend their surgical conference was to share their research through oral or poster presentations. This was in addition to learning about different surgical specialities [13]. We found that keynote speakers were of particular importance as an attraction and valuable element of the conference, emphasising the significance of integrating representation and diversity into the planning of neuroscience conferences. This ensures that the delegates are not only engaged by the scientific topic discussed, but also feel represented.

Our key findings demonstrate positive delegate outcomes by increasing neuroscience career interest, academic skillsets, and career knowledge, supporting the existing single-centre evidence base [7–10]. The illustrated increase in knowledge has been exhibited in further studies showing virtual platforms as a viable educational alternative to traditional in-person teaching, despite some data showing learning objectives are better met via in-person events [14–17]. Interestingly, students did not rank this as a leading factor for their interest in student-led conferences. However, qualitative analysis from our prospective surveys of the cohort demonstrates further development of neuroscience knowledge, suggesting that the neuroscientific themes of the conference remain resonate and perhaps drove an initiative for self-education and research.

The widening global access to technology has allowed for the progressive introduction of virtual components into conferences, with the COVID-19 pandemic further incentivising the movement to online conferences and allowing for their efficacy to be more vigorously scrutinised [18]. In the post-conference group, 65% would prefer neuroscience conferences to be held virtually in the future. In the 6-month follow-up group, the majority (75%) of delegates urged for the continuation of a combination virtual and in-person approach to student-led neuroscience conferencing. Amongst the strengths highlighted by our results and the existing literature, the reduction in cost to both the organisers and the delegates conferred the greatest benefit of an online format. Virtual conferences are cheaper to organise and run, and this combined with the elimination of travel and accommodation fees results in a markedly reduced price of attendance for the delegate [18]. The lowering of costs widens access to those from lower socio-economic backgrounds, allowing for the greater global dissemination of information and opportunities [19]. This is especially relevant considering the majority of our cohort comprises of full-time students who are mostly funded by small means tested loans; however, they may be supplementing this with part time employment or financial support from family members. Nonetheless, it can be argued that moving to virtual conferences merely shifts the barrier of attendance from monetary to technological [20].

Travelling to a scientific conference has been found to constitute 7% of the total yearly CO2 emissions for the average attendee [21]. Switching to a virtual platform for scientific conferences provides the optimum combination of minimal carbon emissions and increased accessibility to disabled individuals or those from a lower socio-economic background [22, 23]. Utilising an online platform is also more convenient, as corroborated by our cohort, because they could attend from the comfort of their home or a place of choice. This point is echoed in the literature where delegates found that they were able to continue daily activities such as their work, home or social life [18]. Furthermore, the option to pre-record lectures allows for presenters from different time zones to still contribute despite the distance [24].

It is important to note that the global transition to online teaching during the COVID-19 pandemic has led to the development of the “Zoom Fatigue” phenomenon. This describes a combination of exhaustion and decreased attentiveness experienced when engaged in long periods of virtual learning and nonverbal overload, such as online conferences [25]. Nevertheless, approximately 6 months post-conference, participants still note the positive influence for online conferences in inspiring an increase in ‘neuroscience career interest’, ‘networking’ and ‘encouragement to participate in research’.

### Future directions

A combined approach constituting in-person and online delivery modes, presents an ideal and viable option for future student neuroscience conferences. Such an approach could be achieved through the filming of an in-person event, offering delegates the option for face-to-face interaction and engagement whilst allowing for a diverse delegate audience with reduced virtual ticket price. This could lead to the inclusion of more attendees from lower socioeconomic backgrounds and international locations, as well as a reduction in CO2 emissions. Practical workshops are often a key component of conferences, as they provide delegates with the opportunity to develop practical skills and use professional equipment. The lack of face-to-face workshops was considered a major drawback by our studied population. Recent advances in simulation technology may more easily allow for the integration of extended reality components into mixed virtual/in-person conferences that attempt to provide a compromise for this drawback [20]. Another benefit of the hybrid approach to be noted is the potential for additional leadership opportunities within the local neuroscience society, providing committee members with additional portfolio enhancement options.

The positive delegates outcomes that were produced consistently across the 6 centres, despite the variety in conference structure and delivery, suggests that conferences focussing on a different medical specialty would produce similar results for delegates. However, further multi-specialty research is required to confirm the generalisability and external validation of our interpretation. More generally given the scarcity of data available on delegate outcomes in medical conferencing, studies are encouraged at student led and postgraduate led level, with aim to provide more effective training to their intended audience.

### Limitations

Our chief limitation is the proportion of participants lost to follow up at 6 months (88.8%), exposing our prospective analysis to a degree of selection bias. The most likely reason our loss to follow up was so high was there was less incentive to fill out the survey at 6 months, given that an automatic attendance certificate link was attached to our post conference survey. It is possible that the narrow cohort of individuals who filled out the 6-month post-conference survey may have been more inclined to do so if they are still interested in a neuroscience career and associated portfolio building activity, and as such may have opened the email from an undergraduate neurological society. Furthermore, improvements following the initial questionnaire should be considered alongside factors such as novelty, excitement, and the Hawthorne effect. Therefore, this prevents us from being able to firmly attain whether longer term results can be attributed to the conference attendance. Furthermore, we do not have a comparison cohort of participants attending in-person neuroscience conferences to determine any potential difference in results as a consequence of mode of delivery. Additionally, some of our multiple-choice options do not offer delegates the opportunity to list their independent answer to the question. However, given the scale, initial piloting of the survey and follow-up re-design, it is likely options would categorise most delegates thoughts. Lastly, from our analysis it is difficult to determine whether the differing conference content and delivery between centres conferred dissimilar benefits without performing a comparison analysis. Nevertheless, our large cohort multi-centre analysis across various virtual conference structures provides evidence to support the continuation of this delivery platform given the bespoke additional offerings this approach holds for delegates and the wider neuroscience community that in-person events cannot sustainably nor exclusively offer.

## Conclusion

Student-led virtual neuroscience conferences in the UK play a role in developing knowledge and may facilitate career interest, academic skillset, and longer-term portfolio building. However, due to the follow up loss at 6 months, other explanations should also be considered, such as perceived improvements in academic skillsets and career interest being short lived and the nature of individuals in the prospective cohort skewing results. This study provides further support for the continuation of student-led virtual neuroscience conferences as a viable alternative to in-person events. Virtual conferences enable a reduced cost to delegates and organisers, widening access for a diverse and inclusive population of delegates and an increased global dissemination of knowledge with a lower carbon footprint. Nevertheless, an ideal solution to future student conferencing would provide delegates with a hybrid virtual and in-person experience, providing options for face-to-face engagement, networking, and interactivity, whilst conferring the benefits of a virtual approach. Future research is encouraged to externally validate our findings and confirm if student-led conferences across a wider range of medical specialties can replicate similar positive outcomes.

### Supplementary Information

**Additional file 1.**

**Additional file 2.**

**Additional file 3.**

**Additional file 4.**

**Additional file 5.**

## Data Availability

The datasets used and/or analysed during the current study are available from the corresponding author on reasonable request.
