# Supplementary material for: Virtual student-led neuroscience conferencing: a UK multicentre prospective study investigating delegate outcomes and delivery mode
Source: BMC Med Educ. 2023 Nov 17;23:883. doi: 10.1186/s12909-023-04779-z (PMC10657021; doi:10.1186/s12909-023-04779-z)
Supplement: Supplementary file 1 — Additional file 1. [file 12909_2023_4779_MOESM1_ESM.pdf]

Pre-Conference Survey

*Required

1. Email *

____

2. What stage are you in training?

Mark only one oval.

- ( ) Medical Student (pre-clinical)
- ( ) Medical Student (intercalating)
- ( ) Medical Student (clinical)
- ( ) Junior Doctor
- ( ) Other: _______________________

3. Have you been to a student led neurosciences conference before?

Mark only one oval.

- ( ) Yes
- ( ) No

4. Are you presenting at this conference?

Mark only one oval.

- ( ) Yes
- ( ) No

5. What attracted you to the conference? (tick all that apply)

Tick all that apply.

- [ ] Opportunity to present
- [ ] Opportunity to network
- [ ] Opportunity to win prizes
- [ ] Opportunity to boost CV
- [ ] Keynote Speakers
- [ ] Other Speakers
- [ ] Workshops
- [ ] Institutions affiliated with our speakers

6. Have you previously participated in any of the following neuroscience career building activities?

Tick all that apply.

- [ ] Neuroscience elective
- [ ] Neuroscience SSC
- [ ] Conference presentation
- [ ] Neuroscience society committee member
- [ ] None of the above

7. Have you undertaken your own neuroscience related research project before?

Mark only one oval.

- ( ) Yes
- ( ) No

8. How interested are you in a neuroscience career? E.g. Neurology, Neurosurgery, Psychiatry...

Mark only one oval.

	1	2	3	4	5	6	7	8	9	10	
Not interested	( )	( )	( )	( )	( )	( )	( )	( )	( )	( )	Complete interest

9. What neuroscience career are you interested in?

Mark only one oval.

- Neurology
- Neurosurgery
- Psychiatry
- Other: ____________________

10. On a scale of 0-10 how prepared do you feel to undertake your own research project?

Mark only one oval.

	0	1	2	3	4	5	6	7	8	9	10	
Not prepared at all												Completely prepared

11. On a scale of 0-10 how prepared do you feel to present at a conference?

Mark only one oval.

	0	1	2	3	4	5	6	7	8	9	10	
Not prepared at all												Completely prepared

12. Do you experience 'Neurophobia'?

Mark only one oval.

- Yes
- No
- Maybe

13. Educational Manipulation Check 1 e.g.
What structures are connected via the indirect subiculothalamic pathway of the Fornix?

Mark only one oval.

- A
- B
- C
- D

14. Educational Manipulation Check 2 e.g.
Which tremor is primarily associated with Parkinsonism?

Mark only one oval.

- A
- B
- C
- D

15. Educational Manipulation Check 3 e.g.
How long is an Academic Clinical Fellowship in Neurosurgery?

Mark only one oval.

- A
- B
- C
- D

16. Educational Manipulation Check 4 e.g.
What is an academic foundation programme?

Mark only one oval.

- A
- B
- C
- D

17. Can we contact you (using the email address collected by this form) in 6-24 months with another survey to complete as part of this study?

Mark only one oval.

- ◯ Yes
- ◯ No

18. What day of the month is your birthday? What are the first three letters of your mother’s first name? This is to ensure we can link responses anonymously through a unique code. E.g. DOB - 14/09/1995. Mother's Name = Caroline. Unique Code = 14Car.

____

This content is neither created nor endorsed by Google.

Google Forms
