# Supplementary material for: Virtual student-led neuroscience conferencing: a UK multicentre prospective study investigating delegate outcomes and delivery mode
Source: BMC Med Educ. 2023 Nov 17;23:883. doi: 10.1186/s12909-023-04779-z (PMC10657021; doi:10.1186/s12909-023-04779-z)
Supplement: Supplementary file 2 — Additional file 2. [file 12909_2023_4779_MOESM2_ESM.pdf]

Post-Conference Survey

1. What was the most valuable part of this conference? (tick all that apply)

Tick all that apply.

- [ ] Opportunity to present
- [ ] Opportunity to network
- [ ] Opportunity to win prizes
- [ ] Opportunity to boost CV
- [ ] Keynote Speakers
- [ ] Other Speakers
- [ ] Workshops
- [ ] Institutions affiliated with our speakers

2. Has this conference inspired you to participate in any of the following?

Tick all that apply.

- [ ] Neuroscience elective/SSC
- [ ] Conference presentation
- [ ] Neuroscience society committee member
- [ ] Neuroscience research project
- [ ] Neuroscience conference
- [ ] Further CV building activity
- [ ] None of the above

3. How interested are you in a neuroscience career? E.g. Neurology, Neurosurgery, Psychiatry...

Mark only one oval.

	1	2	3	4	5	6	7	8	9	10	
Not interested	◯	◯	◯	◯	◯	◯	◯	◯	◯	◯	Complete interest

4. On a scale of 0-10 how prepared do you feel to undertake your own research project?

Mark only one oval.

	0	1	2	3	4	5	6	7	8	9	10	
Not prepared at all	◯	◯	◯	◯	◯	◯	◯	◯	◯	◯	◯	Completely prepared

5. On a scale of 0-10 how prepared do you feel to present at a conference?

Mark only one oval.

	0	1	2	3	4	5	6	7	8	9	10	
Not prepared at all	◯	◯	◯	◯	◯	◯	◯	◯	◯	◯	◯	Completely prepared

6. Educational Manipulation Check 1

Mark only one oval.

- ◯ A
- ◯ B
- ◯ C
- ◯ D

7. Educational Manipulation Check 2

Mark only one oval.

- ◯ A
- ◯ B
- ◯ C
- ◯ D

8. Educational Manipulation Check 3

Mark only one oval.

- A
- B
- C
- D

9. Educational Manipulation Check 4

Mark only one oval.

- A
- B
- C
- D

10. Do you prefer neuroscience conferences to be held online?

Mark only one oval.

- Yes
- No

11. What part of neuroscience conferences being held online do you enjoy the most? (Tick all that apply)

Tick all that apply.

- [ ] Convenience: can access home facilities and resources throughout the day
- [ ] Convenience: does not require you to be formally dressed
- [ ] Convenience: eliminates need for travel
- [ ] No face-to-face interaction
- [ ] No travel costs
- [ ] Flexibility to not attend every talk
- [ ] Increased accessibility internationally

12. What part of neuroscience conferences being held online do you enjoy the least? (Tick all that apply)

Tick all that apply.

- [ ] No opportunity to network
- [ ] Less engagement through online presenting in comparison to face-to-face
- [ ] Lower quality of delivery of online presentations in comparison to face-to-face
- [ ] Home distractions
- [ ] No practical face-to-face workshops
- [ ] No face-to-face interaction with other delegates and presenters
- [ ] No opportunity to travel to a new city
- [ ] Technical Difficulties
- [ ] None

13. What day of the month is your birthday? What are the first three letters of your mother's first name? This is to ensure we can link responses anonymously through a unique code. E.g. DOB - 14/09/1995. Mother's Name = Caroline. Unique Code = 14Car.

____

This content is neither created nor endorsed by Google.

Google Forms
