# Supplementary material for: Virtual student-led neuroscience conferencing: a UK multicentre prospective study investigating delegate outcomes and delivery mode
Source: BMC Med Educ. 2023 Nov 17;23:883. doi: 10.1186/s12909-023-04779-z (PMC10657021; doi:10.1186/s12909-023-04779-z)
Supplement: Supplementary file 3 — Additional file 3. [file 12909_2023_4779_MOESM3_ESM.pdf]

UK Neuroscience Conference 2021 Prospective Survey

1. Which conference(s) did you attend?

Tick all that apply.

- [ ] Sheffield
- [ ] UCL
- [ ] Barts
- [ ] Glasgow
- [ ] Edinburgh
- [ ] Southampton

2. How interested are you in a neuroscience career? E.g. Neurology, Neurosurgery, Psychiatry...

Mark only one oval.

	1	2	3	4	5	6	7	8	9	10	
No interest	○	○	○	○	○	○	○	○	○	○	Complete interest

3. On a scale of 0-10 how prepared do you feel to undertake your own research project?

Mark only one oval.

	0	1	2	3	4	5	6	7	8	9	10	
Not prepared at all	○	○	○	○	○	○	○	○	○	○	○	Completely prepared

4. On a scale of 0-10 how prepared do you feel to present at a conference?

Mark only one oval.

	0	1	2	3	4	5	6	7	8	9	10	
Not prepared at all	○	○	○	○	○	○	○	○	○	○	○	Completely prepared

5. How many presentations have you participated in since the conference?

Mark only one oval.

- 0
- 1
- 2
- 3
- 4+

6. How many neuroscience research projects /audits have you participated in since the conference?

Mark only one oval.

- 0
- 1
- 2
- 3
- 4
- 5
- 6+

7. How else have you developed your neuroscience career interest?

Tick all that apply.

- [ ] Student Selected Component placement (SSC)
- [ ] Elective
- [ ] Neuroscience Conferences
- [ ] Involvement in neuroscience society
- [ ] Teaching
- [ ] Other: ____________________

8. What activities have you confirmed to undergo at a later date?

Tick all that apply.

- [ ] Presentation
- [ ] Research Project
- [ ] Elective
- [ ] Neuroscience conference
- [ ] Neuroscience Society
- [ ] Teaching
- [ ] Other: ______________________

9. What role has the conference played, if any, in facilitating your neuroscience career related activity

____
____
____
____
____

10. Would you like future conferences to be virtual or in person?

Mark only one oval.

- ( ) Virtual
- ( ) In Person
- ( ) Both

11. What day of the month is your birthday? What are the first three letters of your mother’s first name? This is to ensure we can link responses anonymously through a unique code. E.g. DOB - 14/09/1995. Mother's Name = Caroline. Unique Code = 14Car.

____

This content is neither created nor endorsed by Google.

Google Forms
