# Supplementary material for: Virtual student-led neuroscience conferencing: a UK multicentre prospective study investigating delegate outcomes and delivery mode
Source: BMC Med Educ. 2023 Nov 17;23:883. doi: 10.1186/s12909-023-04779-z (PMC10657021; doi:10.1186/s12909-023-04779-z)
Supplement: Supplementary file 4 — Additional file 4. [file 12909_2023_4779_MOESM4_ESM.docx]

**Participant Information Sheet**

The following survey is part of a multi-center prospective study. All responses will be made anonymous. The aim of this voluntary questionnaire is to understand the role of student led virtual conferences. We will be surveying participants before and after the conference to create a comparison of results. Those who consent to follow up will receive a further email at 6-24 months. Please answer the questions honestly. We aim to publish the results in the near future and data will be stored electronically. Queries can be answered by contacting [emily_bligh@hotmail.co.uk](mailto:emily_bligh@hotmail.co.uk). Thank you.

Please note: You may withdraw from the study at any point. Completing the survey/or withdrawing will not impact on your chance of following a neuroscience career pathway.

Will my taking part in this project be kept confidential? All information that is collected about
you during the course of the research will be kept strictly confidential. The information you give will not be used in any way that could identify you and no one outside the research team will know who took part in the survey. Survey responses will be stored separately from email addresses and only those researchers that need to send out the next survey will have access to your email address.

Who is the data controller? The University of Sheffield is the sponsor for this study based in the
United Kingdom. This means that we are responsible for looking after your information and using it properly.

What is the legal basis for processing my personal data? According to data protection
legislation, we are required to inform you that the legal basis we are applying in order to process your personal data is that ‘processing is necessary for the performance of a task carried out in the public interest’ (Article 6(1)(e)). Further information can be found in the University’s Privacy Notice

<https://www.sheffield.ac.uk/govern/data-protection/privacy/general>.
